# Integrative clinical and molecular insights into the comorbidity between depression and sleep apnea syndrome

**DOI:** 10.3389/fpsyt.2025.1659330

**Published:** 2025-10-08

**Authors:** HaiHua Chen, LanMei Zhuang, ZhiJuan Ji, Xing Sun, Ting Zhu, Bo Wang, Jin Wang

**Affiliations:** ^1^ Department of Respiratory Medicine, Shanghai Jing ‘an Shibei Hospital, Shanghai, China; ^2^ Department of Respiratory Medicine, Shanghai Gonghui Hospital, Shanghai, China; ^3^ Australian Chinese Preventive Medicine Association, Sydney, NSW, Australia

**Keywords:** depression, sleep apnea syndrome, adjusted binomial method, pathways, genetic connection

## Abstract

**Objective:**

To identify and characterize overlapping genes and pathways linking Depression and Sleep Apnea Syndrome (SAS).

**Methods:**

A three-level analysis was conducted. Clinically, depression severity in 29 SAS patients was assessed using the Zung Self-Rating Depression Scale. Molecularly, an AI-driven literature mining approach was applied to extract gene–disease associations from PubMed and bioinformatics databases (19,924 genes), with prioritization using the Adjusted Binomial Method and validation via differential expression analysis. Functionally, shared genes were explored through protein–protein interaction (PPI) networks, enrichment analysis, and directional pathway modeling.

**Results:**

Clinically, 62.07% of SAS patients exhibited depressive symptoms, with mild to moderate severity based on the Zung Self-Rating Depression Scale. Molecularly, 872 genes were found to be shared between 4,544 Depression-related and 1,197 SAS-related genes (OR = 11; p = 4.95 × 10^-319^). Further prioritization identified 24 overlapping genes with strong enrichment (OR = 10.9; p = 3.32 × 10^-16^), supported by validation in multiple gene expression datasets. These genes formed a densely connected protein–protein interaction network (238 edges; density = 0.43; clustering coefficient = 0.87), with core hubs including CASP3, TP53, SOD2, HMOX1, and MIR146A. Enrichment analysis highlighted involvement in oxidative stress, ferroptosis, and inflammatory pathways. Directional pathway modeling indicated that SAS may influence Depression via 18 genes and vice versa via 5 genes, with MIF and SOD2 acting as shared regulators.

**Conclusion:**

This study reveals significant clinical and molecular links between Depression and SAS, identifying shared biological pathways and candidate targets for integrated therapeutic strategies.

## 1 Introduction

Depression is a prevalent mental health disorder marked by persistent sadness, loss of interest or pleasure, and a range of cognitive and physical symptoms such as fatigue, sleep and appetite disturbances, and impaired concentration, all of which substantially impair daily functioning (1, 2). Affecting approximately 5% of the global adult population—around 280 million individuals—it occurs more frequently in women than in men (2, 3). Depression often coexists with other mental disorders, including anxiety and substance use disorders (4, 5), as well as with chronic physical illnesses such as cardiovascular disease, diabetes, and cancer, compounding disease burden and worsening health outcomes (6). Similarly, Sleep Apnea Syndrome (SAS), particularly obstructive sleep apnea (OSA), is a prevalent sleep-disordered breathing condition characterized by recurrent upper airway obstruction during sleep, leading to intermittent oxygen desaturation and sleep fragmentation (7, 8). Globally, SAS affects approximately 2–4% of adults, with higher prevalence in men and individuals with obesity or hypertension (7, 8). Untreated SAS could significantly elevate the risk of cardiovascular diseases, including hypertension, stroke, and myocardial infarction (9). These findings are consistently supported by clinical, epidemiological, and guideline-based evidence (7, 9).

Depression and SAS are both prevalent conditions that significantly impact overall well-being, and growing evidence suggests they often co-occur and may exacerbate one another. Emerging research highlights a potential genetic link between the two, indicating that shared genetic factors may contribute to their comorbidity. Genetic predispositions to obstructive sleep apnea (OSA), the most common form of SAS, have been associated with increased risk for a range of health issues, including mood disorders like depression (10, 11). Furthermore, studies have shown a significant association between sleep disorders—including SAS—and depressive symptoms, suggesting a possible bidirectional relationship (12, 13). Genetic polymorphisms, such as those in the MTHFR gene, have also been linked to depression, providing further support for a shared genetic basis (14). These findings underscore the importance of further research into the genetic mechanisms that underlie the co-occurrence of depression and SAS.

Current research has identified genetic factors associated with depressive and SAS independently; however, the shared genetic basis between these two conditions remains largely underexplored. For example, genetic variants such as the A allele of the rs6311 polymorphism have been associated with severe depressive symptoms, indicating a genetic influence on depression (15). Similarly, the severity of OSA, the most common form of SAS, has been linked to altered expression of circadian clock genes, suggesting a genetic component in SAS as well (16). Despite these advances, little is known about the overlapping genetic risk factors between MDD and SAS. This gap underscores the need for further research to uncover potential shared genetic pathways. A better understanding of these shared mechanisms could illuminate the biological underpinnings of their comorbidity and support the development of more targeted and effective therapeutic strategies.

This study investigates the genetic and molecular overlap between Depression and SAS to better understand their comorbidity and uncover shared biological mechanisms. While prior studies have explored each disorder independently, few have systematically addressed their intersection at the molecular level. To fill this gap, we employ a comprehensive, large-scale strategy combining AI-driven literature mining of nearly 20,000 genes, independent validation through gene expression datasets, and advanced network/pathway modeling. This integrative approach allows us to prioritize biologically relevant genes, identify shared pathways, and model potential bidirectional regulatory mechanisms between Depression and SAS. Our findings aim to provide novel insights that support personalized diagnostics and therapeutic development for individuals affected by both conditions.

## 2 Methods and materials

This study employed a three-level analytical framework to investigate the association and shared biological mechanisms between depression and SAS. At the clinical level, we assessed depression severity in SAS patients using the Zung Self-Rating Depression Scale (SDS) to establish the prevalence of comorbid depression. At the molecular level, we performed AI-assisted literature mining and gene expression analysis to identify and prioritize genes associated with both conditions, using the Adjusted Binomial Method (ABM) and differential expression testing. Finally, at the functional level, we conducted enrichment analyses, protein–protein interaction (PPI) network construction, and pathway modeling to explore shared biological functions and mechanisms underlying the observed comorbidity. This multilevel design bridges clinical observations and molecular insights to uncover potential therapeutic targets and biological links between SAS and depression.

### 2.1 Clinical study between SAS and depression

#### 2.1.1 Participant recruitment and eligibility criteria

From 2018 to the present, approximately 500 individuals underwent comprehensive sleep monitoring at our hospital. Among them, 250 participants were diagnosed with SAS, defined by an apnea–hypopnea index (AHI) >5 events/hour. To minimize selection bias and avoid subjective intervention, 40 participants were randomly selected from this eligible pool and invited to complete a depression assessment using the Zung Self-Rating Depression Scale (SDS). After informed consent was obtained, 29 participants returned completed questionnaires and were included in the final analysis. Clinical parameters such as age and sex were recorded and included in the analysis to monitor their potential influence on the results. The study protocol was approved by the Ethics Committee of Shanghai Jing’an Shibei Hospital (Approval ID: 2021422-03).

##### 2.1.1.1 Inclusion criteria

Participants met established diagnostic criteria for SAS, with AHI >15 events/hour; were able to undergo full overnight polysomnography (PSG); and, for those in the intervention group (e.g., receiving BiPAP therapy), adherence was defined as wearing the device for ≥4 hours per night on at least 5 nights per week, accounting for ≥70% of the total treatment time.

##### 2.1.1.2 Exclusion criteria

Individuals were excluded if they had other pulmonary conditions such as pneumothorax, bronchial asthma, pleural effusion, or malignancy; comorbidities severely affecting quality of life including advanced heart, liver, or kidney dysfunction; hematological disorders involving thrombosis, embolism, or coagulation abnormalities; anatomical airway obstructions (e.g., nasal blockage, pharyngeal stenosis, tonsillar hypertrophy, macroglossia) likely to cause snoring or upper airway blockage; contraindications to PSG monitoring; or if they were using long-term sedatives, analgesics, or had psychiatric conditions that could interfere with study participation.

#### 2.1.2 Detection metrics and analysis

Zung’s self-rating depression scale (SDS) served as a direct indicator of patients’ depressive experiences over the week preceding their enrolment in the study (17). The scale comprises 20 items, each rated on a 4-point scale. In this framework, 10 items are scored positively, whereas the other 10 are scored in reverse. The raw score is calculated by summing the scores of all 20 items, and this total is then multiplied by 1.25. The integer part of the resulting product is taken as the standard score. Based on the Chinese norm, a standard score of ≥ 53 indicates depression, with the severity increasing along with the score. More precisely, a score of 53–62 points indicates mild depression, 63–72 denotes moderate depression, and ≥ 73 indicates severe depression.

Measurement data were expressed as the mean ± SE of the mean. Multi-way ANOVA was employed to analyze the risk factors associated with depression in these patients, including Age, Sex, and severity level of Obstructive Sleep Apnea (OSA). A P value < 0.05 was considered statistically significant.

### 2.2 Disease-gene mining and analysis

#### 2.2.1 Disease-gene identification

To identify genes potentially associated with depression and SAS, a large-scale literature mining process was performed, covering 19,924 human genes. Two primary tools were used for data retrieval: the Entrez API (www.ncbi.nlm.nih.gov/Entrez/), which provides automated access to biomedical literature in PubMed, and the AIC Bioinformatics Toolbox (ABT: www.gousinfo.com/en/userguide.html), an AI-powered platform that extracts gene–disease relationships from a proprietary literature database (ABD) as well as public sources like PubMed. Retrieved information—including article titles, publication dates, PMIDs, DOIs, and abstracts—was organized into a structured Excel file for downstream analysis. Both tools enabled efficient large-scale extraction of relevant literature, supporting the identification of candidate genes based on published evidence.

#### 2.2.2 Gene prioritization using adjusted binomial method

To evaluate the reliability of each gene–disease association identified through literature data mining (LDM), we applied the Adjusted Binomial Method Algorithm (ABMA). This statistical approach accounts for both the volume of supporting literature and the consistency (or polarity agreement) of reported associations. The method calculates a confidence score for each gene–disease pair based on its observed literature evidence.

To control for multiple testing and reduce the likelihood of false positives, we applied a False Discovery Rate (FDR) correction, selecting only those associations with an FDR adjusted p-value ≤ 0.05. This filtering step allowed us to retain statistically significant and biologically plausible gene–disease links for downstream functional and network analyses (18).

The statistical significance was assessed using the following formula:


$$\;p-value=\;P\left(X\;\geq \;n_{p}\right)\;=\;binom.sf\left(n_{p}\;-\;1,\;N,\;p_{0}\right)$$

where binom.sf is the survival function of the binomial distribution, $n_{p}$ is the observed number of positive polarity findings, N is the total number of polarity-adjusted observations, and $\;p_{0}$ is the expected null proportion under random association.

#### 2.2.3 Using gene expression data analysis

For the overlapping genes identified through the literature data mining (LDM) process, we conducted an independent gene prioritization step based on gene expression analysis. Specifically, genes were prioritized if they showed statistically significant expression differences (p-value ≤ 0.05 and effect size ≤ -1 or ≥1 ) in any of the selected case–control datasets. This step was independent of the ABM-based literature evaluation.

The selection criteria for gene expression datasets were as follows: 1) The dataset must be based on a case–control study design. 2) Both the expression data file and the corresponding platform annotation file must be fully available for download. **Table 1** lists all the datasets used for the both diseases, including 24 depression datasets and 4 SAS datasets.

**Table 1:** Gene expression datasets used for the gene prioritization.

Disease	GEO list
Depression	GSE114852; GSE12654; GSE32280; GSE35974; GSE35977; GSE35978; GSE39653; GSE44593; GSE45603; GSE46743; GSE53987; GSE54562; GSE54563; GSE54564; GSE54565; GSE54566; GSE54567; GSE54568; GSE54570; GSE54571; GSE54572; GSE54575; GSE92538; GSE98793
Sleep Apnea Syndrome	GSE2271; GSE7224; GSE21409; GSE38792

For each dataset, gene expression levels were log_2_-transformed. One-way ANOVA was then applied to compare the expression levels of each gene between the case and control groups, with both p-values and effect sizes calculated. Given the limited number of genes tested (n = 24), false discovery rate (FDR) correction was deemed unnecessary.

### 2.3 Cross-disease gene analysis

#### 2.3.1 Overlap analysis

Gene lists associated with Depression and SAS were compared to identify both unique and overlapping genes. Fisher’s exact test was applied to evaluate the statistical significance of the observed overlap. To visualize the intersection, a Venn diagram was generated. While comparisons were made using both the full set of disease-associated genes and the subset of prioritized genes, subsequent analyses focused primarily on the prioritized gene set. Notably, for each disease, the prioritized genes included those identified as statistically significant either through ABM analysis or gene expression analysis.

#### 2.3.2 Functional analysis

To better understand the shared biology between depression and SAS, we performed functional annotation and Protein-Protein Interaction (PPI) network analysis on the overlapping genes.

For Functional Annotation, we used the DAVID database (https://david.ncifcrf.gov) to identify enriched biological processes, cellular components, molecular functions (via GO terms: GOTERM_BP_DIRECT, GOTERM_CC_DIRECT, and GOTERM_MF_DIRECT), and known pathways (BBID, BIOCARTA, and KEGG_PATHWAY). This analysis helps reveal the biological roles and pathways these genes are involved in.

For Protein-Protein Interaction (PPI) Network Analysis, we built a PPI network using experimentally validated and literature-supported interactions. We then analyzed the network structure using key topological measures: Network density; Average path length; Clustering coefficient; Diameter; and Centrality measures. Genes that ranked highly across these centrality metrics were considered hub genes, which may play critical roles in the shared disease mechanisms. We further analyzed these hub genes for enriched biological functions.

#### 2.3.3 Pathway analysis

To investigate possible biological pathways connecting Depression and SAS, we constructed a directed gene network based on literature polarity data extracted during the AI-driven literature mining process. Specifically, directional relationships were inferred from curated PubMed abstracts and full texts that reported positive or negative associations between genes and diseases. This polarity information reflects whether the literature supports an upregulating, downregulating, or neutral effect between entities. The directional model was constructed using only statistically significant gene–disease associations (q ≤ 0.05) with clearly defined polarity. The resulting network focused on overlapping genes significantly associated with both Depression and SAS, providing insight into potential bidirectional regulatory mechanisms.

We integrated known or inferred gene-gene interactions to form directional paths—such as depression → Gene A →SAS—which represent potential biological routes through which one disease may influence the other. This network highlights candidate pathways and hub genes that may mediate cross-disease effects and serve as targets for further study.

## 3 Results

### 3.1 Clinical depression assessment in SAS patients

**Figure 1:**
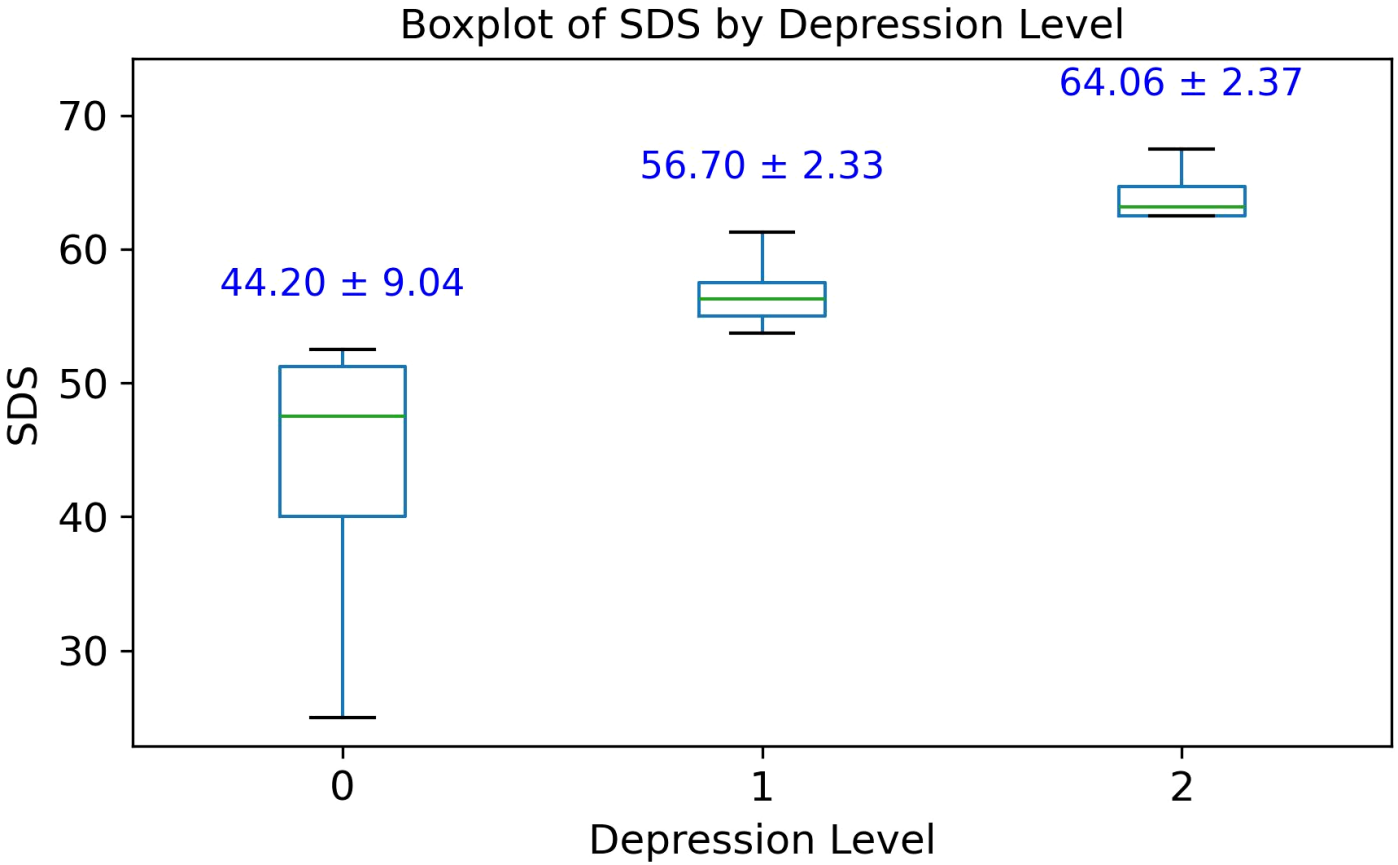
Box plot of Zung self-rating depression scale (SDS) scores among 29 patients with Sleep Apnea Syndrome (SAS). Group 0, no depression; Group 1, mild depression; Group 2, moderate depression.

Out of the 29 patients diagnosed with SAS, 11 (37.93%) exhibited no signs of depression (SDS score: 44.20 ± 9.04), 14 (48.28%) were classified with mild depression (SDS score: 56.70 ± 2.33), and 4 (13.79%) with moderate depression (SDS score: 64.06 ± 2.37), as illustrated in **Figure 1**. No cases of severe depression were observed. Overall, 62.07% of SAS patients demonstrated clinically relevant depressive symptoms, suggesting a substantial comorbidity burden and supporting a potential association between SAS and depression severity.

Multi-way ANOVA analysis was conducted to assess the influence of age, sex, and obstructive sleep apnea (OSA) severity on depression levels among SAS patients. The results showed that neither age (F(15) = 0.941, p = 0.564) nor sex (F(1) = 0.352, p = 0.570) had a statistically significant effect on depression scores (see **Table 2**). OSA severity level demonstrated a marginal association with depression (F(2) = 3.847, p = 0.068), suggesting a possible trend that warrants further investigation with a larger sample size.

**Table 2:** Multi-way ANOVA analysis of risk factors for depression level.

Factors	sum_sq	df	F	PR(>F)
Age	781.430	15.0	0.941	0.564
Sex	19.471	1.0	0.352	0.570
OSA level	426.042	2.0	3.847	0.068
Residual	443.029	8.0	NaN	NaN

### 3.2 Disease-gene identification and comparison results

**Figure 2:**
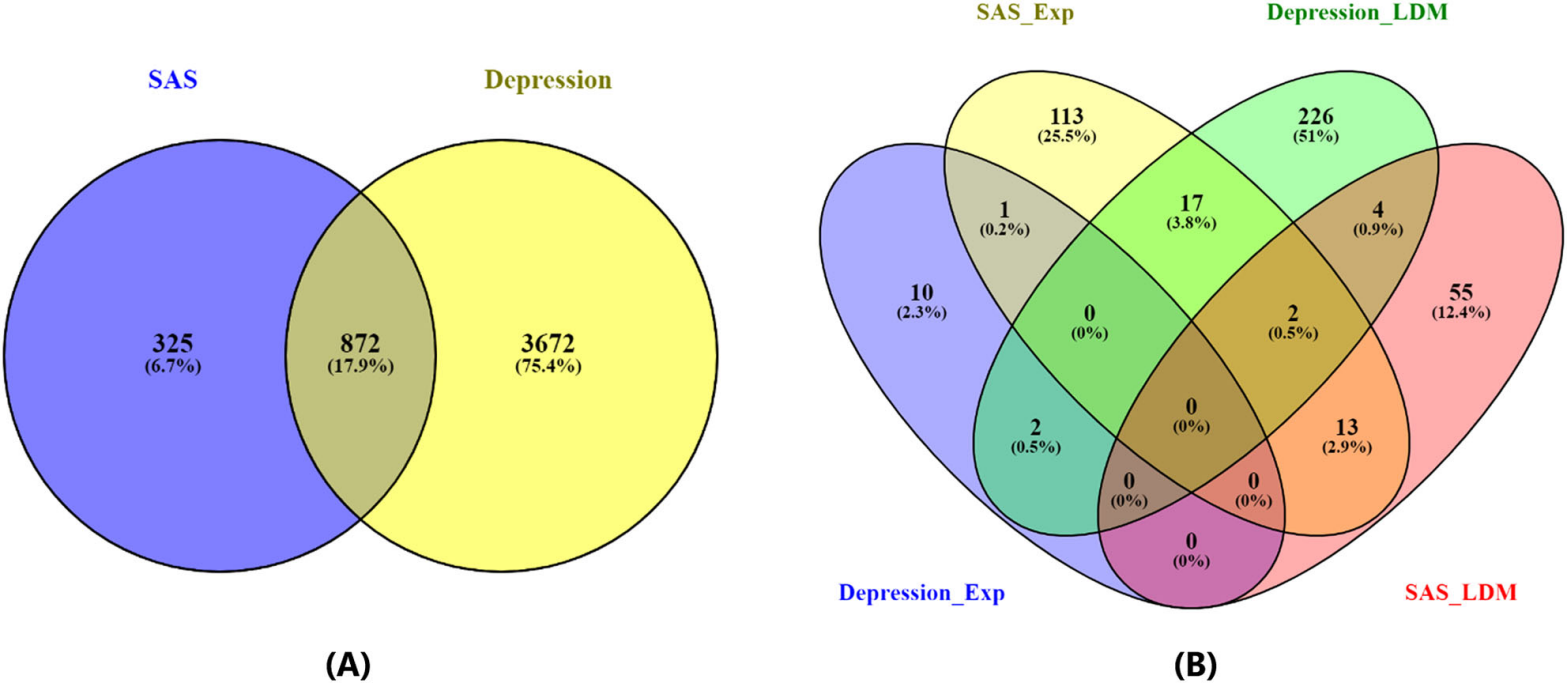
Venn diagrams illustrating the overlap between genes associated with Depression and Sleep Apnea Syndrome. **(A)** Overlap based on all identified disease-related genes. **(B)** Overlap based on statistically significant disease-related genes (p-value ≤ 0.05 for both gene expression analysis and Adjusted Binomial Method (ABM) analysis).

Among a total of 19,924 genes, the AI-driven literature mining approach identified 4,544 genes linked to Depression, supported by 11,141 references (**Supplementary Table S1**), and 1,197 genes linked to SAS, supported by 2,769 references (**Supplementary Table S2**). Of these, 872 genes were shared between the two disorders. As illustrated in **Figure 2A**, this overlap is highly significant, with a Fisher’s exact test revealing an odds ratio (OR) of 11 and a p-value of 4.95 × 10^-319^ (**Figure 2**), indicating a substantial enrichment of common genes between Depression and Sleep Apnea Syndrome.

In addition to the overall gene overlap, a focused analysis on statistically significant genes (p-value ≤ 0.05) revealed 261 genes associated with Depression and 205 genes associated with SAS, with 24 genes overlapping between the two conditions, as shown in **Figure 2B**. Details of gene list in **Figure 2B** are provided in (**Supplementary Table S3**). This overlap demonstrated a strong enrichment, with an odds ratio of 10.9 and a p-value of 3.32 × 10^-16^ (**Table 3**). Notably, the relevance of these genes is further supported by gene expression data: 13 of the 261 Depression-associated genes were validated in three gene expression datasets (GSE182195; GSE54562; GSE54570), while 148 of the 205 SAS-associated genes were confirmed across four datasets (GSE7224; GSE38792; GSE21409; GSE2271), underscoring the robustness of these findings.

**Table 3:** Venn diagram statistics for overlapping genes among three diseases.

Gene Category	Source Disease	Target Disease	#genes Source	#genes Target	Overlap	Odds ratio	p-value
All genes	Depression	SAS	4544	1197	872	11	4.95E-319
Significant Genes (p-value ≤0.05)	Depression	SAS	261	205	24	10.9	3.32E-16

For significant genes, the observed overlap is further supported by gene expression analysis. Specifically, 13 of the 261 Depression-associated genes were validated in three gene expression datasets, while 148 of the 205 SAS-associated genes were validated in four gene expression datasets.

The 24 significant overlapping genes identified for further analysis are: CAMK2B, CASP1, CASP3, CD27, CX3CR1, EGR2, ESR1, FOXP3, GCLC, GLS, GPX4, GSK3B, HGS, HMOX1, MC4R, MIF, MIR146A, NFE2L2, NOD2, SFN, SOD2, TET1, TNXB, and TP53, as detailed in the subsequent sections.

Many of these overlapping genes have well-established roles in biological processes relevant to both Depression and SAS. For instance, TP53 is a master regulator of cellular stress responses, including apoptosis and oxidative stress, which are commonly dysregulated in neuropsychiatric and metabolic disorders. CASP3, a central executioner in the apoptotic pathway, is implicated in neuroinflammation and neuronal cell death, processes shared by both conditions. Other genes such as SOD2 and GPX4 are involved in antioxidant defense mechanisms, while MIR146A regulates immune responses. These examples illustrate that the shared gene set is not only statistically enriched but also biologically meaningful, supporting the hypothesis of converging molecular pathways in Depression and SAS.

### 3.3 PPI analysis

**Figure 3:**
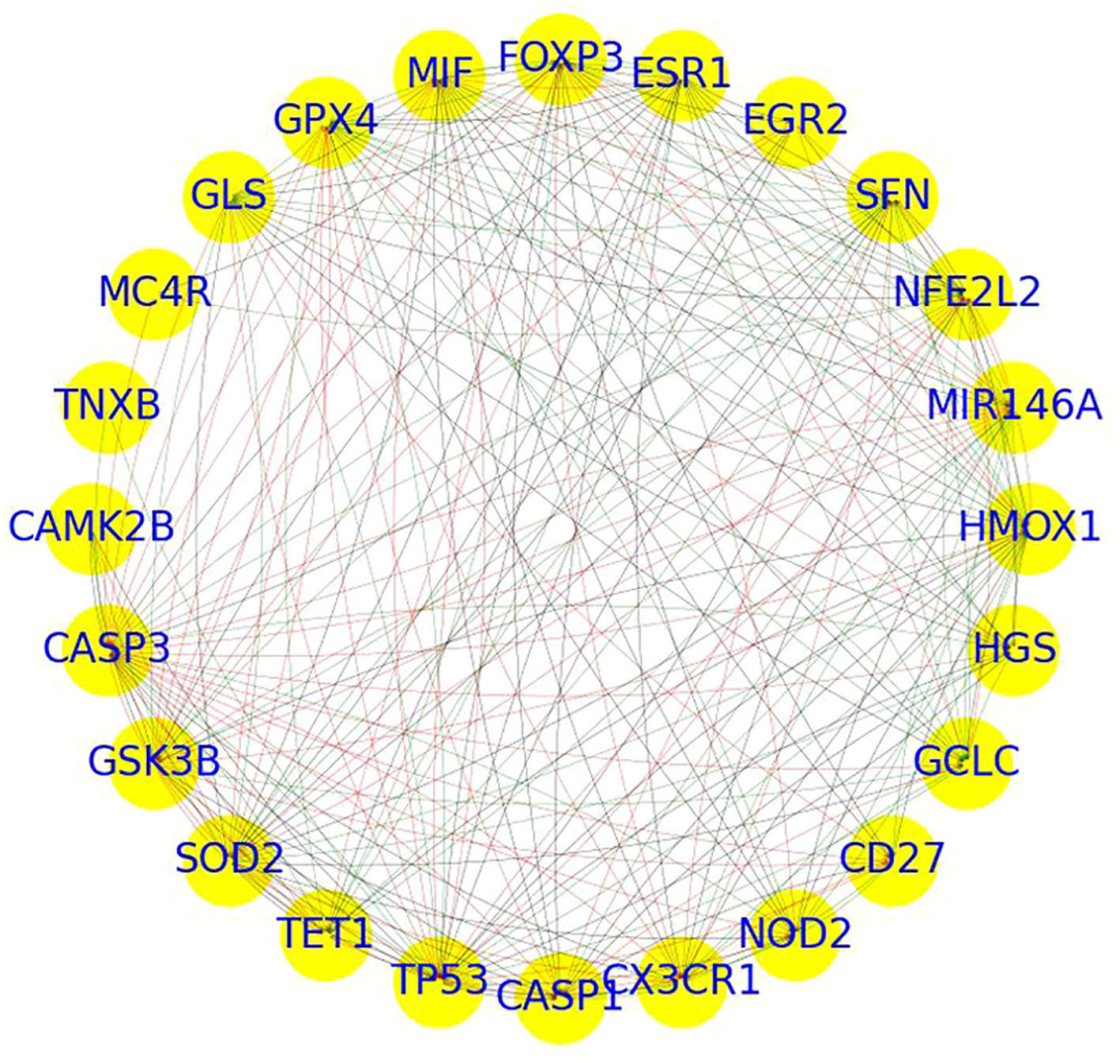
PPI network of overlapping genes between depression and sleep apnea syndrome.

PPI network analysis of 24 genes revealed a highly dense and cohesive network connected by 238 edges (**Figure 3**). The network exhibited a high density of 0.43, a short average path length of 1.52, a clustering coefficient of 0.87, and a diameter of 2, indicating a compact structure with substantial interconnectivity. The network formed a single connected component, underscoring a tightly integrated module potentially involved in shared molecular functions.

Centrality analysis identified several hub genes with prominent network roles.

- CASP3, TP53, HMOX1, NFE2L2, and SFN displayed high in-degree centrality (≥0.65), suggesting they are key recipients of interactions.
- Genes such as SOD2, TP53, MIR146A, FOXP3, GPX4, MIF, and GLS exhibited high out-degree centrality (≥0.6), indicating broad regulatory influence.
- Although betweenness centrality values were generally low, CASP3 (0.09), TP53 (0.07), and SOD2 (0.05) stood out as potential signal mediators within the network.
- Eigenvector centrality highlighted CASP3, TP53, SOD2, GPX4, GSK3B, and HMOX1 (≥0.24) as influential nodes connected to other highly connected genes.

Together, these metrics designate CASP3, TP53, SOD2, HMOX1, and MIR146A as core hub genes, likely contributing to the functional backbone of the network. Further pathway and functional enrichment analysis of these key genes is presented in the following section.

### 3.4 Functional annotation analysis results

To explore the biological functions of the 24 significant overlapping genes between Depression and SAS, we performed functional enrichment analysis (as described in the Methods).

**Figure 4:**
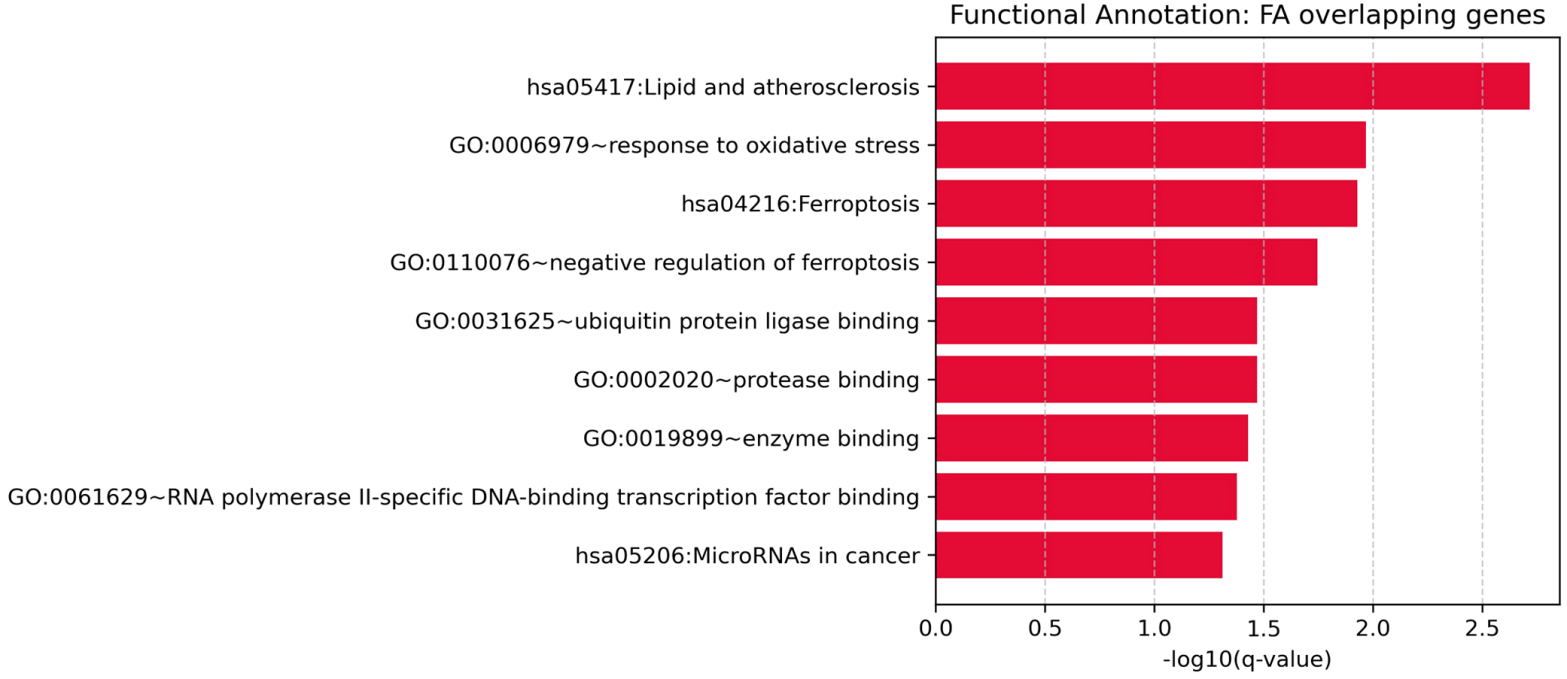
Functional enrichment analysis for overlapping genes associated with both depression and sleep apnea syndrome.

This analysis revealed nine significantly enriched pathways or functional categories (**Figure 4**), primarily related to oxidative stress, ferroptosis, and cancer-associated processes. Key terms included “response to oxidative stress” (GO:0006979, Bonferroni-adjusted p = 0.0108), “ferroptosis” (KEGG hsa04216, Bonferroni-adjusted p = 0.0237), and “lipid and atherosclerosis” (KEGG hsa05417, Bonferroni-adjusted p = 0.00195). Several genes—such as TP53, NFE2L2, HMOX1, GPX4, and GSK3B—were involved in multiple enriched terms, suggesting their key roles in stress response and disease-related pathways.

### 3.5 Pathway connecting depression and SAS

**Figure 5:**
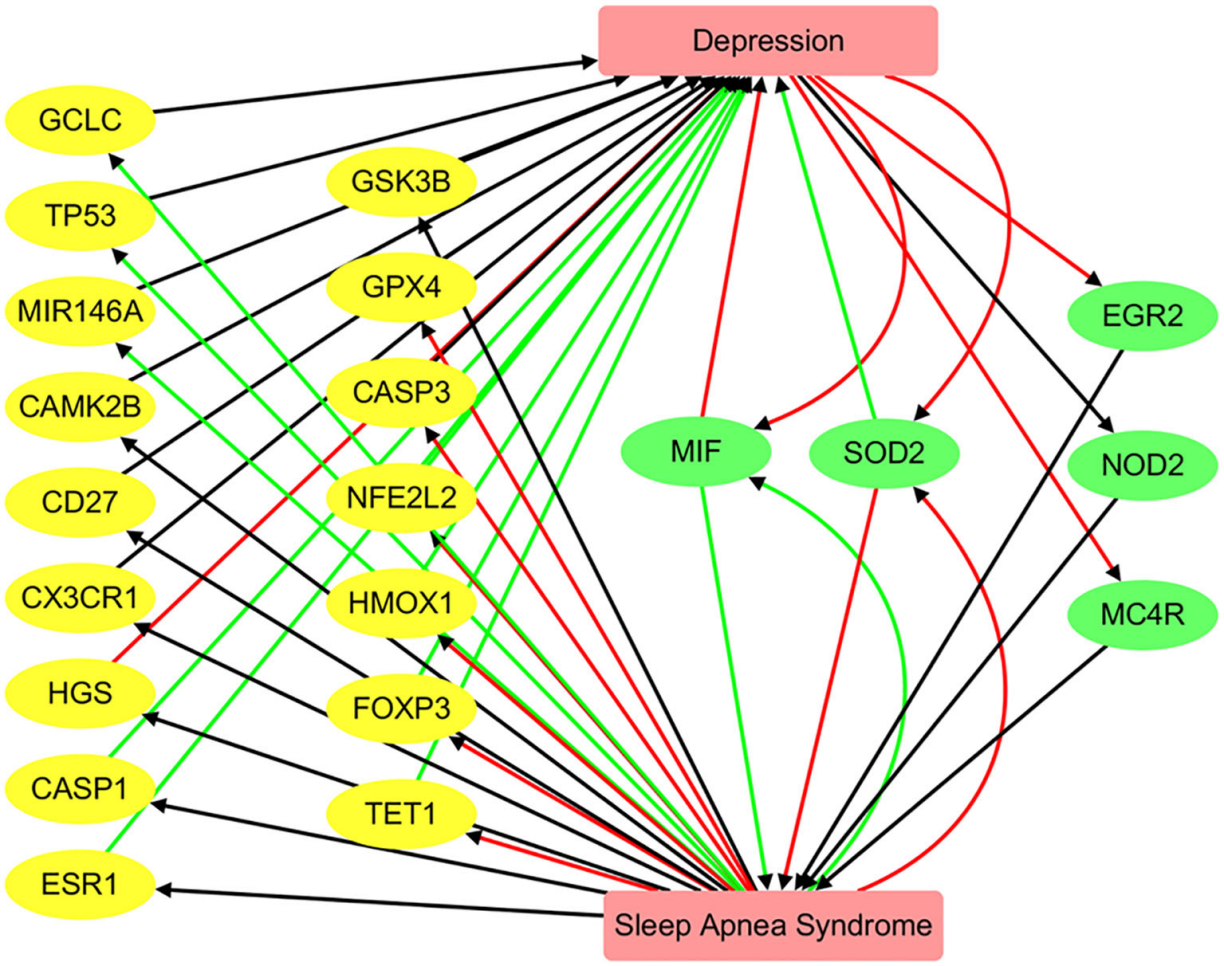
Pathway connecting depression and sleep apnea syndrome. Green edges represent positive associations, red edges indicate negative associations, and black edges denote associations with unknown polarity.

Functional pathway analysis revealed a bidirectional regulatory relationship between Depression and SAS through a shared set of functionally relevant genes (**Figure 5**). Depression positively regulates key genes such as NFE2L2 (10 references; q = 0.0052) and HMOX1 (3 references; q = 0.0326), both of which are involved in oxidative stress responses. Depression also negatively regulates EGR2 (3 references; q = 0.0326), MC4R (3 references; q = 0.0326), and SOD2 (5 references; q = 0.0036), indicating its suppressive effect on metabolic and redox-related pathways.

These genes, in turn, are significantly altered in SAS. For example, FOXP3 (9 references; q = 0.00033), SOD2 (9 references; q = 0.00033), and GPX4 (4 references; q = 0.0061) are strongly downregulated in SAS, while MIF is highly upregulated (8 references; q = 3.32 × 10^-5^). This pattern suggests that SAS and Depression influence a common network of genes that mediate inflammation, oxidative stress, and immune regulation.

The reciprocal regulation of genes such as MIF, SOD2, and GPX4 supports the existence of a Depression → gene → SAS feedback mechanism, where Depression-driven gene dysregulation may contribute to SAS pathophysiology, and vice versa. The consistent polarity and significant q-values observed across these pathways highlight shared molecular mechanisms between the two disorders. However, we emphasize that these should be interpreted as hypothesis-generating and not as definitive mechanistic pathways. Experimental validation is required to confirm these proposed gene-mediated influences.

## 4 Discussion

This study provides integrated clinical and molecular evidence supporting a strong bidirectional relationship between Depression and SAS. Clinically, 62.07% of SAS patients exhibited depressive symptoms, reinforcing their comorbidity. Molecularly, AI-assisted literature mining identified 872 genes shared between the two conditions—a highly significant overlap (odds ratio = 11, p = 4.95 × 10^-319^) that aligns with prior associations between sleep disturbances and mood disorders (10, 11). The 24 statistically prioritized overlapping genes, especially TP53, CASP3, SOD2, HMOX1, and MIR146A, formed a tightly connected protein interaction network and were enriched in pathways related to oxidative stress, ferroptosis, and inflammation. These hub genes are known mediators of cellular stress, apoptosis, and immune regulation, suggesting they may serve as molecular links between SAS-related hypoxia and depression-related neuroinflammation. While previous research has explored the relationship between Depression and various other disorders (19, 20), as well as the association of SAS with conditions such as asthma (21), our study is the first to systematically investigate the molecular and clinical link between Depression and SAS.

Our study primarily focuses on the association between Depression and SAS at the genetic level. As illustrated in **Figure 5**, SAS may influence the pathology of Depression through the regulation of 18 genes, while Depression may affect SAS via 5 genes. Notably, MIF and SOD2 are shared between the two conditions. These genes form a significantly interconnected network, as revealed by protein–protein interaction (PPI) analysis, with central hub genes identified as CASP3, TP53, SOD2, HMOX1, and MIR146A. These findings represent plausible directional hypotheses derived from curated gene–disease polarity data, and future experimental work is needed to test these relationships.

While a detailed discussion of each gene’s role is beyond the scope of this section, we highlight CASP3 and TP53 as representative examples. In SAS, chronic intermittent hypoxia—a hallmark of obstructive sleep apnea (OSA)—has been shown to increase levels of cleaved caspase-3, promoting neuronal apoptosis and cognitive impairment, thereby implicating CASP3 in the pathological effects of SAS on the brain (22). Likewise, in major depressive disorder (MDD), CASP3 has been identified as a key therapeutic target, with molecular docking studies supporting its role in neuroprotection and antidepressant effects (23). This dual involvement suggests that CASP3 may act as a molecular bridge linking SAS and depression. Similarly, TP53, a gene central to apoptosis and oxidative stress, appears to be another shared molecular mediator. Multiple studies have identified TP53 as a core target involved in depression pathophysiology and treatment response [PMID: 40010035]. For instance, network pharmacology analyses revealed TP53 as a central node modulated by traditional Chinese medicines such as Xiaoyao pills, baicalin, and Danzhixiaoyao pills, which exert antidepressant effects possibly through PIK3/AKT signaling and apoptosis-related pathways [PMID: 40010035]. In SAS, chronic intermittent hypoxia upregulates p53 expression, inducing vascular endothelial cell senescence and contributing to vascular aging and cardiovascular risk [PMID: 38168028]. Moreover, SAS triggers apoptosis in neuronal and myocardial tissues via caspase activation [PMID: 39694586, 37037067], implicating upstream regulators such as TP53. Together, these findings support a model in which SAS may contribute to depression by modulating TP53- and CASP3-mediated apoptotic pathways.

An association between Depression and SAS is also evident at the cell process level, involving interconnected inflammatory, oxidative, and hormonal mechanisms. For example, intermittent hypoxia in SAS triggers systemic inflammation and oxidative damage, which in turn may exacerbate depressive symptoms (24). Additionally, GSK3B links mood regulation and metabolic processes, suggesting its role in hormonal and stress-related dysfunction (25). Genetic predisposition further contributes to this comorbidity; variants in genes involved in inflammatory and stress-response pathways are enriched in individuals with both disorders (21), and immune system genes activated by hypoxia are implicated in sustained inflammation in SAS (26). Hormonal dysregulations—particularly involving cortisol and leptin—also bridge the two conditions by affecting both mood and sleep regulation (27). Altogether, these findings suggest that the cellular-level interplay among oxidative stress, immune activation, genetic susceptibility, and hormonal imbalance forms the mechanistic basis of the comorbidity between MDD and SAS.

An association between Depression and SAS is also evident at the organ and tissue level, involving the brain, cardiovascular system, and respiratory system. In the brain, MDD regulates genes such as NFE2L2 and HMOX1, which are crucial for mitigating oxidative stress, while SAS influences genes like FOXP3, SOD2, and GPX4, suggesting a shared molecular network that contributes to neuroinflammation and altered neurotransmitter function. This interaction may underlie the cognitive and emotional symptoms observed in both conditions, further supported by evidence that SAS disrupts brain network connectivity and respiratory regulation (10). In the cardiovascular and respiratory systems, SAS leads to intermittent hypoxia and sympathetic nervous system activation, increasing the risk of hypertension, ischemic heart disease, and cardiac remodeling—pathways that are also influenced by MDD through chronic inflammation and oxidative stress (28). Similarly, in the lungs, SAS contributes to inflammation and fibrosis via chronic intermittent hypoxia, processes that may be exacerbated by MDD-related neuroimmune dysregulation (29). Adipose tissue also plays a key role in the MDD–SAS connection, as obesity—common in SAS—is associated with systemic inflammation and metabolic disturbances that can intensify depressive symptoms (30). Altogether, the convergence of dysregulated processes across the brain, heart, lungs, and adipose tissue highlights a complex, systemic interaction between MDD and SAS, underscoring the importance of integrated therapeutic strategies that target these interconnected organ systems and molecular pathways.

This study leverages an AI-driven literature mining approach combined with rigorous statistical filtering via the Adjusted Binomial Method (ABM) to identify a significant overlap of genes associated with Depression and SAS. The ABM accounts for both the volume and polarity consistency of literature evidence, enhancing confidence in gene–disease associations. The integration of independent gene expression analysis across multiple case–control datasets provides an additional layer of validation, increasing the robustness and biological plausibility of the findings. Furthermore, PPI network analysis reveals a highly interconnected module of core hub genes, and functional enrichment analysis identifies key shared pathways—such as oxidative stress and ferroptosis—offering a comprehensive understanding of the molecular mechanisms linking the two disorders.

Despite these strengths, this study has several important limitations. First, the clinical findings are based on a relatively small cohort (n = 29), which limits statistical power and may reduce the generalizability of the observed comorbidity between Depression and SAS. As such, the clinical results should be interpreted as preliminary and hypothesis-generating, pending validation in larger and more diverse populations. Second, the molecular findings rely on large-scale literature-based mining, which is inherently constrained by the scope, quality, and reporting biases of the available published literature. Although the Adjusted Binomial Method (ABM) and independent gene expression datasets were employed to improve robustness, the literature-derived associations are limited to gene-level links and may not reflect upstream regulatory dynamics, epigenetic influences, or non-coding RNA involvement. Third, while our pathway and network analyses provide insight into potential shared mechanisms, the directionality inferences are based on literature polarity and not on direct mechanistic experiments. Furthermore, none of the identified hub genes or pathways have been experimentally validated in this study.

To strengthen biological relevance and clinical applicability, future research should incorporate *in vivo* or *in vitro* functional experiments—such as animal models or gene perturbation studies—to validate key genes and pathways. Additionally, longitudinal clinical cohorts and intervention-based studies (e.g., CPAP therapy trials in SAS patients with depressive symptoms) could clarify causal relationships. Integrating multi-omics data, including transcriptomics, epigenomics, and metabolomics, may also provide a more comprehensive understanding of the biological interface between Depression and SAS.

## 5 Conclusion

This study provides integrated clinical and molecular evidence supporting a strong comorbidity between Depression and SAS. By identifying shared genes, enriched biological pathways, and polarity-informed directional associations, our findings offer novel hypotheses regarding their interconnected pathophysiology. While these results are not yet sufficient to inform clinical practice directly, they highlight candidate molecular targets and mechanisms that merit further investigation. These insights lay a foundation for future studies—such as functional validation, multi-omics integration, and clinical trials—that may ultimately guide the development of more personalized approaches to diagnosis and treatment.

## Data Availability

The raw data supporting the conclusions of this article will be made available by the authors, without undue reservation.

## Supplementary material

Supplementary Table 1 Relationship table of depression-associated genes. This table lists genes associated with depression, including supporting evidence from the literature and/or databases used in the study.

Supplementary Table 2 Relationship table of sleep apnea syndrome (SAS)-associated genes. This table provides the gene associations identified for SAS, along with annotations or literature evidence supporting these relationships.

Supplementary Table 3 Gene lists used for the VENN plot in **Figure 2B**. Contains the sets of genes associated with depression, SAS, and their overlap, used to generate the Venn diagram presented in **Figure 2B**.
